# Impact of a DSS-supported medication review on the safety of drug therapy and quality of life in patients with antithrombotic therapy

**DOI:** 10.3389/fphar.2024.1194201

**Published:** 2024-05-23

**Authors:** Tanja Elnaz Hassanzadeh, Carina Hohmann, Carsten Culmsee

**Affiliations:** ^1^ Pharma4u GmbH, Munich, Germany; ^2^ Institute for Pharmacology and Clinical Pharmacy, Faculty of Pharmacy, University of Marburg, Marburg, Germany; ^3^ Department of Pharmacy, Klinikum Fulda gAG, Fulda, Germany

**Keywords:** drug safety, medication review, community pharmacy, interprofessional collaboration, polypharmacy, quality of life, antithrombotic medication

## Abstract

Polypharmacy is common among patients with antithrombotic medication, giving rise to concerns about Drug-Related Problems (DRPs). Therefore, these patients would benefit from a Medication Review (MR) along with pharmacist counselling to reduce the risks accompanying polymedication. This prospective study presents a concept for MRs that are applicable in German community pharmacies and can efficiently support pharmacist counselling and improve the safety of drug therapy. As this is a major challenge in everyday pharmacy practice, we used a Decision Support System (DSS) to evaluate its ability to support the process of pharmacist-led MRs. The primary endpoint was the impact of a community pharmacist on the reduction of DRPs. We investigated the impact of the interventions resulting from MRs on patients taking at least one antithrombotic drug as part of their polymedication regimen. Secondary endpoints were the reduction in the number of patients with bleeding risks and the improvement of patients’ Quality of Life (QoL) and therapy adherence. Furthermore, the DSS used in the study was controlled for correct data assessment and plausibility of data. We selected adult patients who were taking no less than three different medications for long-term treatment, at least one of which had to be an antithrombotic drug, and who were customers in one of eight selected pharmacies over a period of 6 months. Data from 87 patients were analyzed with DSS-support. A total of 234 DRPs were identified by the pharmacist (2.7 DRPs per patient). MR reduced DRPs by 43.2% which, resulting to a reduction of 1.2 DRPs per patient. The intervention also led to a significant improvement in the patients’ QoL (assessed via EQ-5D-5L questionnaire; *p* < 0.001) and enhanced therapy adherence (assessed via A14 questionnaire; *p* < 0.001). The control of correct data assessment (with 93.8% concordance) and plausibility of data (with 91.7% concordance) of the DSS software were conducted by an external auditor. No significant effect was found for overall bleeding risk. The results of this study indicate that DSS-supported and structured MR conducted by pharmacists can contribute to a reduction in DRPs and significantly improve patient’s QoL and adherence to treatment.

## 1 Introduction

The QoL of patients taking antithrombotic drugs can be compromised by both their underlying health condition and their medication. Vitamin K antagonists (VKAs) inhibit the synthesis of coagulation factors in the liver, which usually lasts for several days. In addition, the VKA blood levels are affected by numerous interactions (food containing vitamin K, pharmacokinetic drug interactions via competition in the CYP or pGP system, or effects on plasma protein binding). In contrast, Direct Oral Anticoagulants (DOACs) are more selective and interfere with the coagulation cascade via direct thrombin inhibition or factor Xa inhibition. Due to their shorter Half Life (HL) and fewer interactions, DOACs are considered to have a better safety profile than VKAs. Additionally, antidotes such as andexanet alfa and idarucizumab counteract the adverse effects of DOACs more rapidly. Furthermore, the lack of correlation between INR levels and the efficacy of DOACs eliminates the practical need for routine monitoring of coagulation parameters (Pirlog AM et al., 2019). This aspect is particularly important, as it may negatively affect factors that influence patient adherence and control of antithrombotic therapy (Davis et al., 2005; Raparelli et al., 2017; Sridharan et al., 2020). Frequent doctor appointments for regular checks, such as an INR blood test or therapeutic drug monitoring might be perceived as stressful and time-consuming by some patients. In contrast, an increase in the subjective feeling of having control over one’s own disease could help patients to deal with their situation and thus improve adherence and QoL at the same time. Good examples of this are self-monitoring programs for patients with chronic anticoagulation which were carried out in several projects. Most of these studies have shown that self-monitoring can improve clinical outcomes as well as adherence, patient satisfaction and QoL (Levi, 2008; McCahon et al., 2011; Siebenhofer et al., 2012; Soliman Hamad et al., 2009; Solvik et al., 2019; Verret et al., 2012). However, several risk factors for poor anticoagulant status were identified in a study of 15.834 self-monitoring patients taking VKAs, showing that self-monitoring may be useful for most, but not all patients (Schaefer et al., 2016). Since 2002, pharmacists in Germany have established their role in clinical pharmacy and it is also included in the curricula for pharmaceutical education at German universities (Dircks et al., 2017). Several studies have shown that pharmacists can contribute to improving the medication process of patients with different diseases (Rotta et al., 2015), and in the case of antithrombotic patients, could minimize risks by increasing adherence through education and, thereby, raising awareness to the risks of bleeding or inadequate efficacy.

In addition, drug interactions in polypharmacy are very common in elderly patients who often suffer from multiple comorbidities. Especially in patients taking antithrombotics, drug interactions could cause serious unwanted side effects or, on the contrary, destroy the efficacy of drug therapy (Schneider et al., 2018). In fact, bleeding risks are among the five most frequent causes of hospitalization (Thürmann and Schmiedl, 2011). A recent study of elderly patients discharged from hospital showed that the prescription of multiple drugs, potentially causing DRPs, occurred in almost two-thirds of patients (Carpenter et al., 2019). Pharmacists could be in the best position to detect possible DRPs in patients who receive prescriptions from different specialists and general practitioners or additional drugs by self-medication in the community pharmacy. Therefore, pharmacists could significantly help to increase the safety of drug therapy in patients with polypharmacy where there may be a communication gap between the different treating health specialists (Rankin et al., 2018; Dias et al., 2019; Georgiev et al., 2019; Kasper et al., 2020). MRs led by pharmacists are considered an effective instrument in establishing the safety of drug therapy and QoL for the patients (Bulajeva et al., 2014).

Austin et al., 2020 provided a meta-analysis of 27 studies involving patients on anticoagulant treatment concerning the influence of an Electronic Medical Record (EMR) and a DSS on medication errors, DRPs, patient outcomes, quality use of anticoagulants, patient adherence and cost effectiveness. The results from this study suggest that computerized physician order entry (CPOE) in conjunction with DSS might help to effectively manage therapeutic anticoagulation. But the authors found that most research evaluating MRs in patients on oral anticoagulants has focused on prescribing and documentation of adherence, with less focus on the actual clinical impact on patients. Further, most of the previous studies conducted in hospitals lack impact for ambulant and interprofessional medication settings in community pharmacies. So far, interventional studies addressing routine care of patients under antithrombotic therapy with the aid of a DSS have not been reported in community pharmacies in Germany to date.

DRPs are events or circumstances during drug therapy that actually or potentially prevent the achievement of intended therapeutic goals (Phamaceutical Care Network Europe, 2017). In our study, we investigated the effects of pharmacist-led and digitally supported MRs involving bleeding risks, QoL, adherence and the influence on reducing DRPs in the ambulant setting. The main objective of the current study was to improve the safety of drug therapy and the QoL in patients with a minimum of three different drugs containing at least one antithrombotic. We wanted to find out how pharmacists can contribute to improve the safety of drug therapy for these patients in community pharmacies under daily routine conditions. Thus, we examined the effects of interventions through MRs on DRPs, such as undesirable side effects, drug interactions and the extent to which the number of DRPs could be reduced. Further, we analyzed the effects of a pharmacist-led MR on the QoL of the patients, the number of patients with bleeding risks and on the patient´s adherence.

Interdisciplinary cooperation in MRs can be of great advantage to improve the safety of drug therapy of patients with polymedication (Köberlein-Neu et al., 2016). Since 2012, in accordance with the German Ordinance on the Operation of Pharmacies (ApBetrO §1a), MRs should be offered as a service, however, they are still rarely practiced in the day-to-day business of community pharmacies (Schindler et al., 2020a). One important reason for this is, among others, that until very recently, health insurance funds did not cover the costs for a MR or other related medication management activities.

## 2 Materials and methods

### 2.1 Study design

The prospective interventional study was conducted between February 2019 and February 2020. The primary endpoint was the impact of a community pharmacist on the reduction of DRPs. We investigated whether the interventions resulting from MR affected patients taking at least one antithrombotic drug as part of their polymedication regimen. Secondary endpoints included the reduction in the number of patients with bleeding risks and the improvement of patients’ QoL and therapy adherence. In addition, controls were performed for correct data evaluation (validation) and plausibility checks (verification) of the software MediCheck (pharma4u GmbH) which was used as the DSS for this study.

The sample calculation was based on the study by Sennesael et al., 2018 and coworkers, which examined clinical bleeding complications in 46 DOAC and 43 VKA patients.

### 2.2 Patients (inclusion criteria)

For the study, adult patients (≥18 years of age) who were being treated with at least three different drugs prescribed by a physician for long-term treatment (longer than 28 days) were enrolled by the eight participating community pharmacies in Munich. At least one drug had to be an antithrombotic drug (such as VKA, DOAC, heparins or platelet-inhibitors). Patients with end-stage chronic kidney disease (CKD5) who required dialysis (CKD stage 5D) were excluded from the study.

### 2.3 Endpoints and study instruments

#### 2.3.1 DRP analysis, assisted by DSS

All MRs were digitally supported using the DSS MediCheck (pharma4u GmbH). The software program captures all relevant and available patient data, current medication, diseases, laboratory values and current symptoms of the patients via the input mask. The result of the analysis was divided into the so-called “green light check,” which confirms optimal therapy standards (no DRP detected automatically), and the “red light check” showing all detected DRPs. These results are displayed according to prioritization and recommendation in the output mask of the software. MediCheck automatically searches for the following DRPs: side effects, interactions, contra-indications, geriatric suitability (according to PRISCUS and FORTA), dosage of drugs (also in case of renal insufficiency), overuse (medicines without indication), underuse (indication without medication), guideline conformity, prescription cascades, double and pseudo-double medication, nutritional correlation, divisibility. Additionally, MediCheck supports the assessment of adherence by determining whether medication consumption aligns with the prescribed regimen. In addition, the software examines whether routine monitoring is recommended for the current medication and provides disease-related recommendations for prevention. All DRPs found in this study are stated in Table 2 and further specified in Table 3. The type and number of checks corresponds to a type 3 MR according to the ABDA classification (ABDA, 2018). The DRP classification and coding were conducted with the harmonized PCNE scheme, which was divided into “Problems,” “Causes” and “Interventions” with “Communication” and “Plans” (Phamaceutical Care Network Europe, 2017; adapted). The PCNE-Codes have been extended and modified to provide a more comprehensive description of DRPs and were functionally integrated into the software. The assessment of the clinical-pharmaceutical relevance of the results was carried out by the conducting pharmacist, who determined the relevant DRPs from the total number of possible DRPs. The selection of DRPs deemed relevant was based on the severity of the detected DRPs and the acute problems and symptoms described by the patient.

#### 2.3.2 Implementation concept in community pharmacies

For the selection of the eight community pharmacies, we looked for a cooperation partner where the conducting pharmacist could lead the study. That is why the study took place in the “Bienen” pharmacies in the Munich area, where a representative cross-section of pharmacy types, consisting of inner-city pharmacies, suburban pharmacies and rural pharmacies was guaranteed under the same franchise cooperation. The conducting pharmacist was an employee of these pharmacies, which is necessary for legal reasons in order to be able to carry out the MRs of the patients’ medication in compliance with legal standards in Germany. A concept for the implementation of the MR process in these pharmacies was developed prior to the start of the intervention and was divided into the following steps:

1. To fulfill the inclusion criteria, patient records stored within the pharmacy software were filtered based on the following parameters: at least one antithrombotic drug with ATC code level B01 and a minimum of three drugs as long-term medication. In total, more than 500 patients were identified and marked in the pharmacies’ software. In case of a visit of the patients concerned, the pharmacy staff personally informed them about the opportunity to participate in the study and explained the details of the legal requirements. Therefore, all employees of the eight participating pharmacies were actively involved in personally motivating the patients to get enrolled. To this end, the pharmacy staff were trained by the conducting pharmacist on the process of standardized patient outreach and recruitment so that the counselling was harmonized across all participating pharmacies. Patients were required to sign a written informed consent form for data storage and an informed consent for participation in the study. This additional consent form also included a confidentiality agreement between the treating physicians and the pharmacy staff. The MR procedure was based on the guidelines of the German Pharmacists Association (ABDA, 2018). After a period of 1 year, 113 patients provided their written consent to participate. This ensured the sample size calculation, and the recruitment phase was completed. After successful recruitment, a preliminary medication list was generated from the patients’ customer history in the pharmacy computer system.
2. In an initial interview (brown bag consult) lasting approximately 45 min, all relevant baseline data were recorded, including three questionnaires to record QoL, bleeding risk and therapy adherence. Personal data, medication including dosage, indication/diagnosed diseases, clinical or vital parameters and problems or symptoms were then analyzed in detail with IT support from the DSS, automatically searching for the DPRs as formerly described. The data were collected from medication lists, physicians’ and hospital discharge letters. If the data were not available in this form, patients’ statements were recorded if they knew their diagnoses. Laboratory parameters were documented only if a blood test result was accessible from the physician’s office. All steps of the MR (except patient recruitment) were always carried out independently by the same conducting pharmacist. A review of the MR process was then carried out by an external auditor. Detected DRPs and suggestions for changes to the medication plan were communicated to the attending physician, if necessary. A final meeting (baseline) followed within 4 weeks after the brown bag consult, where all DRPs and solutions were explained to the patient. Also, an optimized medication plan was created with IT support and handed out to the patient. This finalized medication plan contained corrected and up-to-date information about dosages, instructions for use and diagnosis.
3. A structured follow-up interview took place between one and 6 months later. Any DRPs were reassessed and discussed with the patient and with the physician, if necessary. During this follow-up interview, all previously completed questionnaires were once again filled out by the patient. All data were analyzed and compared with the data of the initial assessment.

#### 2.3.3 Questionnaires and classification of interventions

Three different questionnaires were used in the study to assess the defined endpoints:

The evaluation of the patients’ QoL was carried out using the short form of the European Questionnaire of Quality of Life with five dimensions and five levels (EQ-5D-5L), which was originally developed by the EuroQol Group (EuroQol Research Foundation, 2019). The EQ-5D-5L includes the 5 dimensions “mobility,” “self-care,” “usual activities,” “pain/discomfort,” and “anxiety/depression.” Each dimension contains five response options: “no problems,” “slight problems,” “severe problems,” “unable to/extreme problems.” The descriptive EQ-5D-5L system includes a total of 3125 distinct health states, each denoted by a unique 5-digit numerical combination. A single summary index can be created from the individual health states using a set of values based on the general population. An index score of “1” indicates the best possible health value, while lower values are associated with deficits in health status. The second part of the questionnaire (the Visual Analogue Scale, EQ VAS) captures the patient’s perception of their general health status: 100 equals “best health you can imagine” and 0 corresponds to “worst health you can imagine”.

The influence of the MR on bleeding risks was recorded using the HEMSTOP questionnaire (Bonhomme et al., 2016), a 7-point questionnaire, each with a response option of “yes,” “no,” or “no answer”. The determined HEMSTOP-score (1 score for each question answered with “yes”) was compared, with 2 or more positive responses indicating an elevated risk of bleeding. Bleeding risks were ascertained, i.e., hematoma, hemorrhage, menorrhagia (women), surgery, dental extraction, obstetrics (women), parents (hereditary coagulation disease).

Therapy adherence was evaluated using the A14 Questionnaire for assessment of adherence and individual barriers (Jank et al., 2009). The A14 scale consists of 14 questions with responses given using a five-point Likert scale from “never” (4 points) to “very often” (0 points). A total score is calculated from the 14 questions with a minimum of zero points and a maximum of 56 points. Patients are categorized as “adherent” (score ≥50 points) or “non-adherent” (score <50 points).

All three questionnaires were completed twice by each patient: the first time within the initial interview and the second time within the follow-up assessment between one and 6 months later. Thus, the information provided by the patients prior to the MR served as a comparative collective to evaluate the questionnaires.

Furthermore, the PCNE scheme (Phamaceutical Care Network Europe, 2017; adapted) was used to qualitatively differentiate whether the DRP was related to antithrombotic or co—medication, and to quantify the extent to which the changes to the medication plan recommended by the pharmacist were implemented by the patient and/or the physician.

#### 2.3.4 Correct data assessment and plausibility of data

The applied MediCheck DSS software was controlled for correct data assessment (validation) and plausibility of data (verification) by an external auditor who had the required qualifications (e.g., license to practice, experience in clinical pharmacy and MRs). For this purpose, 30 from the overall 87 patient cases of the study were selected at random (Number Generator, 2022). For correct data assessment (validation): 1. All documented patient data (date of birth, age, sex, vital signs, renal values), 2. Medication data (trade names, active ingredients, quantities, dosages, type of application, indications if applicable), 3. Diseases (diseases, allergies, ICD-10 codes if applicable), 4. Laboratory and vital signs (vital signs, units, reference ranges, date information), 5. Problems/symptoms (problems, symptoms, terms or technical terms indicated) were checked for plausibility.

The following five questions were addressed for each individual DRP: 1. In which validated source is the selected DRP verified? 2. Is the content of the DRP technically correct, according to the source? 3. Does the context (of the DRP chosen to be relevant) plausibly match the overall case? 4. Are the PCNE codes given and documented correct? (PCNE version 8.0, adapted). 5. Are the selected drugs correctly documented and plausible?

These 30 randomly selected patients had a total of 90 DRPs that were assessed as relevant. For each 90 DRPs, all the above-mentioned items (900 in total) were checked by the auditor. Thus, 450 items each were checked for plausibility and correctness.

#### 2.3.5 Financial compensation

In this study, the pharmaceutical service was offered to patients as a free service. All entities participating in the study (pharmacists, patients, physicians, institutions, and companies) cooperated voluntarily and without any remuneration for their contributions.

### 2.4 Data analysis and statistics

All data were anonymized for the evaluation process. PASS 14 software was used to calculate the sample size (PASS, 2018). Statistical analyses were made using SPSS Statistics version 28 (IBM Corp, Armonk, NY, United States). The Kolmogorov-Smirnov test was used to test for normal distribution of the sample. The *t*-test for dependent samples was used to determine the differences in the frequency of DRPs before and after the MR (Field, 2013). The asymptomatic 2-tailed Wilcoxon rank sum test was used to evaluate QoL and therapy adherence. The Chi-Square Independence Test was used to assess bleeding risk. *p* values <0.05 were considered as significant in all tests (Cohem, 1988).

### 2.5 Ethics approval

Before starting patient recruitment, the project outline was submitted to the responsible ethics committee. The result of the ethics committee’s assessment was unambiguous with the confirmation that no ethics vote would be required if the patients signed a written declaration of consent for data storage in the pharmacy and a declaration of consent to participate in the study. This additional informed consent form also contains an agreement to release confidentiality between the treating physicians and the pharmacy.

## 3 Results

### 3.1 Analysis of drugs used

Among a total of 113 potentially eligible patients, who met all inclusion criteria, 87 underwent a MR and could be assessed within the study. Participation involved 40 (46%) men and 47 (54%) women, with an average recruitment age of 71 (+/− 14.2).

A total of 623 different drugs were prescribed to the 87 patients, classified by ATC-level 2 (WHO) with the most frequent drug classes antithrombotics, medication for lipid metabolism, drugs influencing the renin-angiotensin-aldosterone system (RAAS), ß-adrenoceptor antagonists and pain medication. A complete overview of all drug classes taken by the patients in the current study have been listed in detail (see Supplementary Material). Altogether, 94 antithrombotic drugs were registered as permanent medication. Among these, 73.5% of the patients were treated with monotherapy and 17.3% received a combined antithrombotic therapy. Acetylsalicylic acid (ASA) was the most common medication as a monotherapy. Eight patients who had been prescribed an anticoagulant had usually discontinued their medication on their own. An outline of the mono- and combination therapy of the antithrombotic medication taken by our study subjects (excluding the 8 patients mentioned above) is listed in Table 1.

**TABLE 1:** Number and frequency of the antithrombotic drugs as mono- and combination-therapy.

ATC-code level	Substance class	Number of patients	Frequency (%)
	Monotherapy		
B01AC06	Acetylsalicylic Acid (ASA)	37	42.5
B01AF03	Edoxaban (DOAC)	4	4.6
B01AF01	Rivaroxaban (DOAC)	4	4.6
B01AF02	Apixaban (DOAC)	9	10.3
B01AA04	Phenprocoumon (VKA)	8	9.2
B01AB13	Certoparin 8.000 (Heparin)	1	1.2
B01AC04	Clopidogrel (P_2_Y_12_-Antagonists)	1	1.2
	**Combination Therapy**		
B01AC06 + B01AF01	ASA + Rivaroxaban	1	1.2
B01AC06 + B01AF02	ASA + Apixaban	4	4.6
B01AC06 + B01AA04	ASA + VKA (Phenprocoumon)	1	1.1
B01AC06 + B01AC04	ASA + Clopidogrel	5	5.7
B01AC06 + B01AC24	ASA + Ticagrelor	1	1.1
B01AC06 + B01AC22	ASA + Prasugrel	1	1.1
B01AF02 + B01AA04	Apixaban (DOAC) + Phenprocoumon (VKA)	1	1.1
B01AF03 + B01AF03	Edoxaban (DOAC) + Enoxaparin 10.000 (Heparin)	1	1.1
	Patients who stopped taking their antithrombotic medication	8	9.2

### 3.2 Identification and reduction of DRPs

#### 3.2.1 Number of identified DRPs

**FIGURE 1:**
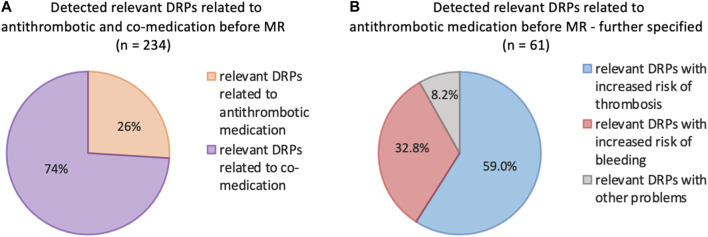
**(A)** Frequencies of relevant DRPs related to antithrombotic or co-medication of all detected relevant DRPs (*n* = 234) before MR. **(B)** Further specified frequencies of DRPs identified as relevant according to the antithrombotic medication; patients with at least 3 permanent medications, at least one of which was an antithrombotic (*n* = 87).

In the first analysis (pre-intervention) at least one DRP was detected in 80 (92%) of the 87 patients. In total 234 DRPs were discovered, which corresponds to a mean of 2.7 DRPs per patient. Figure 1 points out the DRPs before MR related to antithrombotic and co-medication **(A)**, DRPs further specified related to antithrombotic medication **(B)**.

#### 3.2.2 Classification of interventions

Detailed evaluation of the interventions and their relation between antithrombotic and co-medication are listed in Tables 2–5.

**TABLE 2:** DRPs identified using PCNE (adapted).

P-code	Problem domain	Related to Co-Medication	Related to antithrombotic medication	Total
**P 2.1**	Problem with drug safety	133 (56.8%)	41 (17.5%)	174 (74.4%)
**P 1.2**	Drug not optimal (effect)	23 (9.8%)	8 (3.4%)	31 (13.3%)
**P 1.3**	Untreated indication (underuse)	5 (2.1%)	7 (3.0%)	12 (5.1%)
**P 3.4**	Problem (other)	11 (4.7%)	3 (1.3%)	14 (6.0%)
**P 3.2**	Unnecessary drugs (overuse)	1 (0.4%)	2 (0.9%)	3 (1.3%)
**P-Codes**	**Total**	**173 (73.9%)**	**61 (26.1%)**	**234 (100%)**

The bold values represent the total numbers/percentages.

**TABLE 3:** Causes identified using PCNE (adapted).

C-code	Causes domain	Related to Co-Medication	Related to antithrombotic medication	Total
**C 1.4**	Inappropriate drug combination/interaction	51 (21.8%)	32 (13.7%)	83 (35.5%)
**C 8.2e**	Suspicion: drug-related side effect/adverse drug reaction	53 (22.7%)	8 (3.4%)	61 (26.1%)
**C 1.1e**	Contraindication (diseases)	12 (5.1%)	0	12 (5.1%)
**C 1.6**	Indication without drug (underuse)	3 (1.3%)	8 (3.4%)	11 (4.7%)
**C 1.1**	Drugs not in compliance with guidelines	6 (2.6%)	5 (2.1%)	11 (4.7%)
**C 1.4c**	Medication Cascade	10 (4.3%)	0	10 (4.3%)
**C 2.1a**	Dosage form not suitably divisible	8 (3.4%)	1 (0.4%)	9 (3.9%)
**C 3.5**	Intake time error	5 (2.1%)	0	5 (2.1%)
**C 8.2a**	Problems due to illness	3 (1.3%)	2 (0.9%)	5 (2.1%)
**C 3.1**	Underdosed	4 (1.7%)	0	4 (1.7%)
**C 3.2**	Overdosed	4 (1.7%)	0	4 (1.7%)
**C1.1a**	Not a drug of choice according to the guideline	1 (0.4%)	1 (0.4%)	2 (0.9%)
**C 1.3**	No indication (overuse)	0	2 (0.9%)	2 (0.9%)
**C 1.5b**	Pseudo double medication (group)	1 (0.4%)	1 (0.4%)	2 (0.9%)
**C 6.1**	Drugs administered incorrectly- timing	3 (1.3%)	0	3 (1.3%)
**C 7.1**	Fewer drugs than prescribed - Patient	2 (0.9%)	1 (0.4%)	3 (1.3%)
**C 7.7**	Taking time error - patient	2 (0.9%)	0	2 (0.9%)
**C 1.1c**	Unsuitable (PRISCUS)	1 (0.4%)	0	1 (0.4%)
**C 1.1h**	Contraindication (age)	1 (0.4%)	0	1 (0.4%)
**C 3.5c**	Intake meal wrong	1 (0.4%)	0	1 (0.4%)
**C 7.7a**	Wrong intake meal - patient	1 (0.4%)	0	1 (0.4%)
**C 8.2b**	Problems due to laboratory value deviation	1 (0.4%)	0	1 (0.4%)
**C-Codes**	**Total**	**173 (73.9%)**	**61 (26.1%)**	**234 (100%)**

The bold values represent the total numbers/percentages.

**TABLE 4:** Interventions (communication and plan) using PCNE (adapted).

I-code	Intervention domain (communication)	Related to co-Medication	Related to antithrombotic medication	Total
**I 2.3**	Patient is referred to doctor	112 (47.9%)	31 (13.3%)	143 (61.1%)
**I 2.1**	Patient counseling	30 (12.8%)	21 (9.0%)	51 (21.8%)
**I 0.1**	No intervention	14 (6.0%)	4 (1.7%)	18 (7.7%)
**I 2.2**	Patient is advised in writing	13 (5.6%)	5 (2.1%)	18 (7.7%)
**I 2.4**	Info to family member/caregiver	3 (1.3%)	0	3 (1.3%)
**I 1.1**	Doctor is only informed	1 (0.4%)	0	1 (0.4%)
**I-Codes**	**Total**	**173 (73.9%)**	**66 (28.2%)**	**234 (100%)**

I-Code	Intervention Domain (plan)	Related to co-Medication	Related to antithrombotic Medication	Total
**I 4.1**	Other intervention	40 (17.1%)	12 (5.1%)	52 (22.2%)
**I 3.1**	Change dosage (to…)	44 (18.8%)	3 (1.3%)	47 (20.1%)
**I 3.4**	Change instructions (to…)	24 (10.3%)	22 (9.4%)	46 (19.7%)
**I 3.6**	Prepare new drugs	11 (4.7%)	10 (4.3%)	21 (9.0%)
**I 0.1**	No concrete suggestion	15 (6.4%)	5 (2.1%)	20 (8.6%)
**I 3.5**	Discontinue drugs	3 (1.3%)	16 (6.8%)	19 (8.1%)
**I-Codes**	**Total**	**160 (68.4%)**	**74 (31.6%)**	**234 (100%)**

The bold values represent the total numbers/percentages.

**TABLE 5:** Acceptance levels using PCNE (adapted).

A-code	Acceptance domain	Related to Co-Medication	Related to antithrombotic medication	Total
**A 1.1**	Accepted: fully implemented	71 (30.3%)	33 (14.1%)	104 (44.4%)
**A 3.1**	Communicated: Acceptance questionable	65 (27.8%)	17 (7.3%)	82 (35.0%)
**A 3.2**	Proposal not communicated	15 (6.4%)	5 (2.1%)	20 (8.6%)
**A 1.4**	Accepted: Implementation questionable	7 (3.0%)	0	7 (3.0%)
**A 2.2**	Not accepted: no consent	5 (2.1%)	1 (0.4%)	6 (2.6%)
**A 2.4**	Not accepted: unknown	5 (2.1%)	1 (0.4%)	6 (2.6%)
**A 1.3**	Accepted: not implemented	1 (0.4%)	2 (0.9%)	3 (1.3%)
**A 1.2**	Accepted: partially implemented	1 (0.4%)	2 (0.9%)	3 (1.3%)
**A 2.1**	Not accepted: not possible	2 (0.9%)	1 (0.4%)	3 (1.3%)
**A-Codes**	**Total**	**172 (73.5)**	**62 (26.5%)**	**234 (100%)**

The bold values represent the total numbers/percentages.

#### 3.2.3 Reduction of DRPs

**FIGURE 2:**
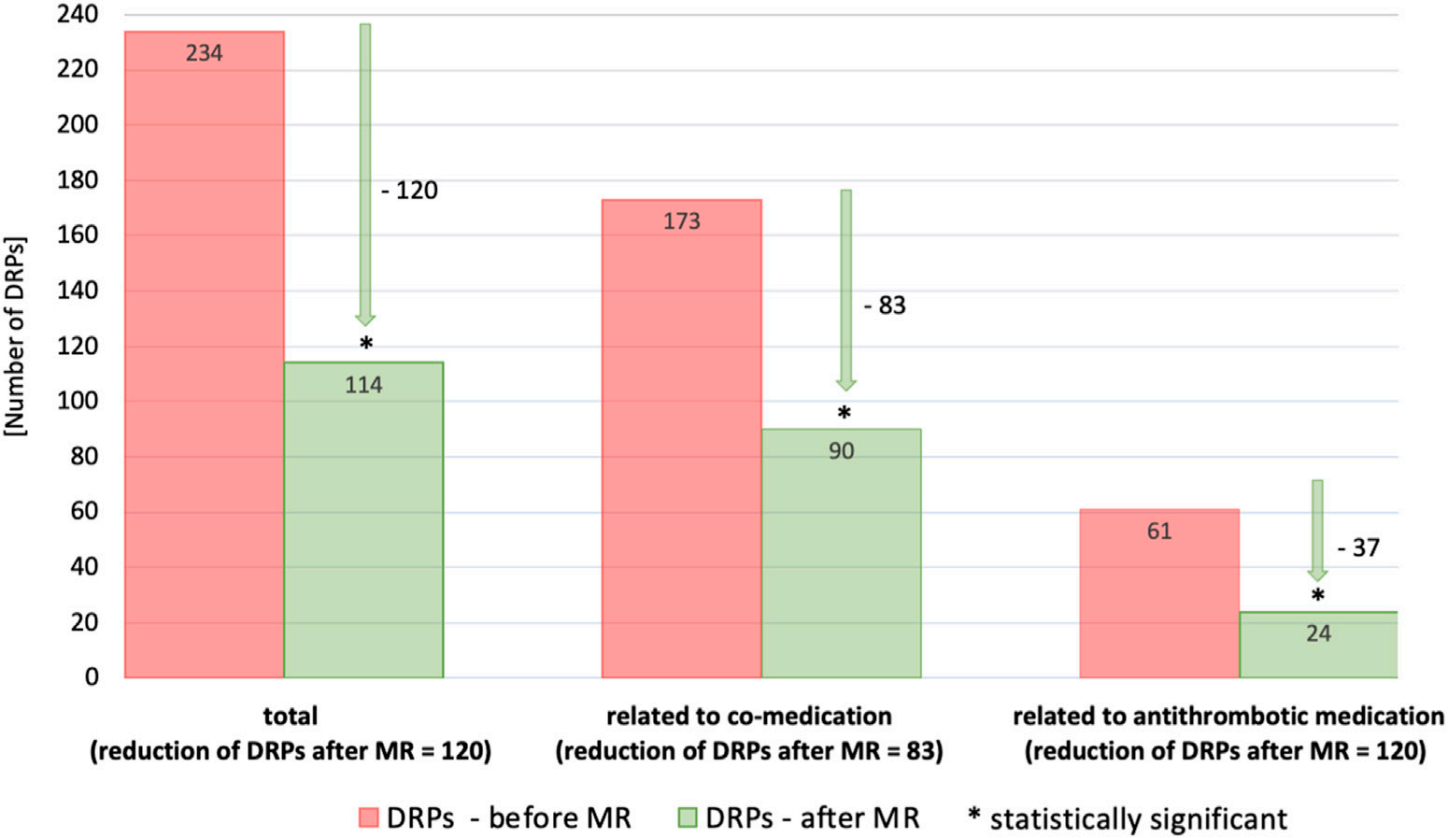
Number of DRPs identified as relevant before and after MR documented with PCNE (total, related to co-medication, related to antithrombotic medication). Statistical significance was calculated using *t*-test for dependent samples (*p* < 0.001); patients with at least 3 permanent medications, at least one of which was an antithrombotic (*n* = 87).

**FIGURE 3:**
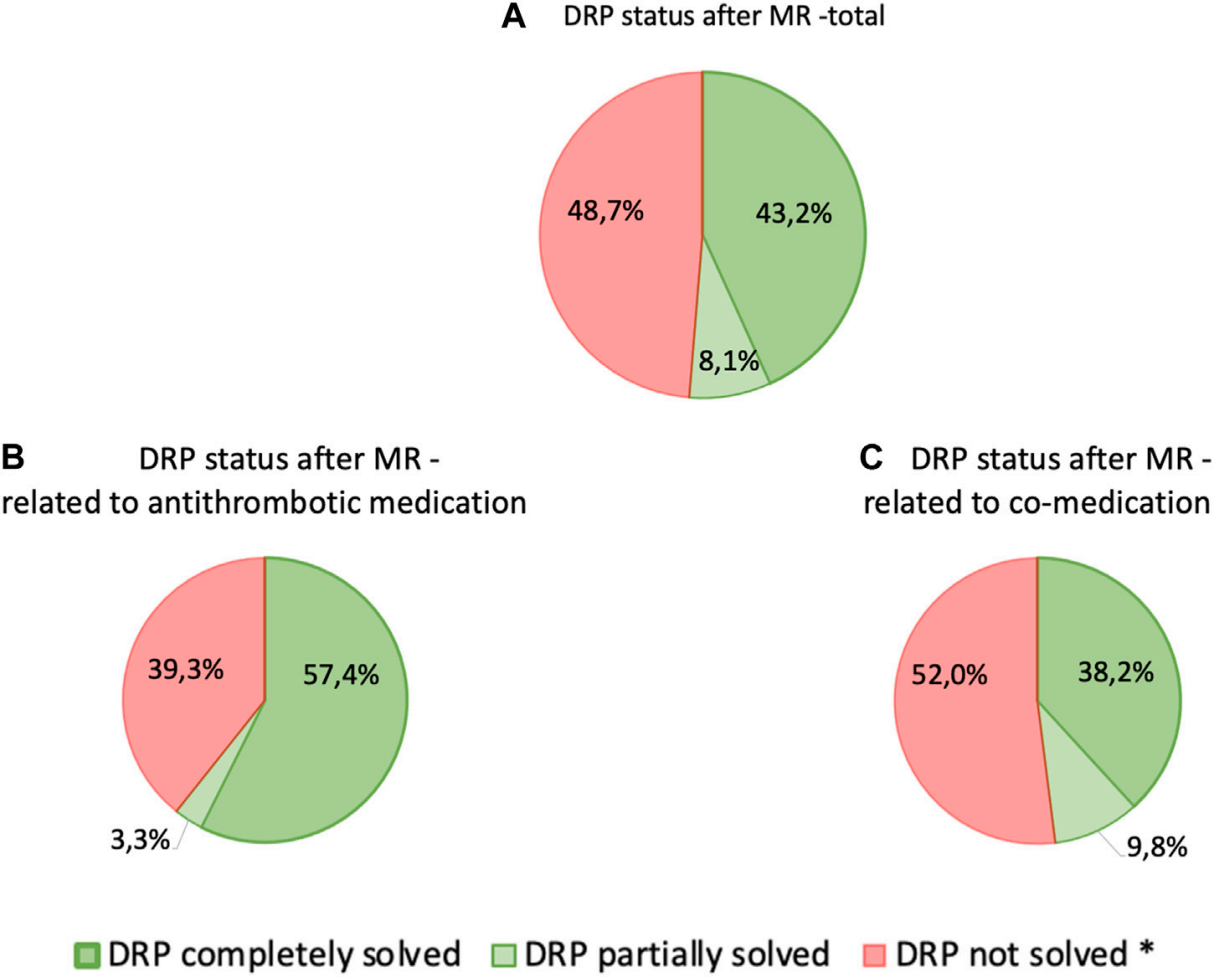
Status of relevant DRPs after completion of MR classified according to PCNE—**(A)** total DRPs, *n* = 234, **(B)** DRPs related to antithrombotic medication, *n* = 61, **(C)** DRPs related to co-medication, *n* = 173; patients with at least 3 permanent medications, at least one of which was an antithrombotic (*n* = 87) * Includes: “status DRP unknown,” “problem solution not necessary/possible,” “DRP not solved, doctor uncooperative,” “DRP not solved, patient uncooperative”.

Post-intervention results were investigated one to 6 months later during the follow-up interview. Detailed information about the reduction of DRPs through our intervention is provided in Table 6 as well as in Figures 2, 3.

**TABLE 6:** DRP Status using PCNE (adapted).

O-code	Final status domain	Related to Co-Medication	Related to antithrombotic medication	Total
**O 1.1**	DRP completely solved	66 (28.2%)	35 (15.0%)	101 (43.2%)
**O 0.1**	Status DRP unknown	60 (25.6%)	11 (4.7%)	71 (30.3%)
**O 3.4**	Problem solution not necessary/possible	22 (9.4%)	8 (3.4%)	30 (12.8%)
**O 2.1**	DRP partially solved	17 (7.3%)	2 (0.9%)	19 (8.1%)
**O 3.2**	DRP not solved, doctor uncooperative	6 (2.6%)	5 (2.1%)	11 (4.7%)
**O 3.1**	DRP not solved, patient uncooperative	2 (0.9%)	0	2 (0.9%)
**O-Codes**	**Total**	**173 (73.9%)**	**61 (26.1%)**	**234 (100%)**

The bold values represent the total numbers/percentages.

### 3.3 Quality of life

**FIGURE 4:**
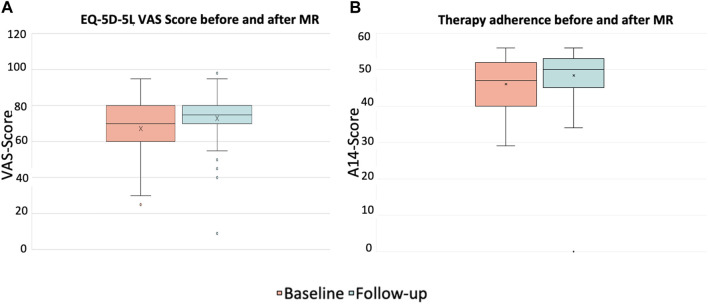
EQ-5D-5L VAS values for self-reported health status **(A)** and therapy adherence **(B)** before and after the intervention (*p* < 0.001, asymptotic 2-tailed Wilcoxon rank sum test); patients with at least 3 permanent medications, at least one of which was an antithrombotic (*n* = 87).

The evaluation of the EQ-5D-5L questionnaire to assess QoL revealed that through a MR performed by the pharmacist and subsequent consultation the patients’ QoL had significantly improved (*p* < 0.001) in both areas analyzed. First, the descriptive part shows a statistically significant improvement of the index value from M = 0.81 (SD = 0.2) to M = 0.88 (SD = 0.1), second, the evaluation of the VAS improved from M = 67.3 (SD = 14.4) to 73.0 (SD = 13.4). These results are shown in Figure 4A.

### 3.4 Therapy adherence

The evaluation of the A14 questionnaire to assess patients’ adherence to therapy showed significant improvement from M = 46.0 (SD = 7.4) before the intervention to M = 48.4 (SD = 7.6) after the intervention (*p* < 0.001), see Figure 4B.

### 3.5 Bleeding risk

The study aimed to assess whether the pharmacist´s intervention had an impact on bleeding risk, evaluated using the HEMSTOP questionnaire. An analysis utilizing a Chi-Square Independence Test was performed to compare the questionnaires before and after the intervention. The results indicated no statistically significant differences, as the independence hypothesis was rejected. The cross-tabulation showed minimal to no differences, and the test discarded the notion of independence.

### 3.6 Control for correct data assessment and plausibility of data

The evaluation of the control for correct data assessment (validation) and plausibility of the data (verification) of the DSS software MediCheck showed consistencies in the pharmacists’ evaluation of DRPs. There was a total of 900 items, 450 each for correct data assessment (validation), of which 259 items could be evaluated by the auditor with 93.8% agreement and 450 items for plausibility of data (verification) with 374 items that could be rated with 91.7% concordance.

## 4 Discussion

The results of our study show that patients receiving antithrombotic drugs are often multimorbid patients with a high number of DRPs. As shown in the current study, many of these DRPs result from co-medication, confirming the findings of previous similar studies on DRPs due to polymedication. For example, Lenssen et al. (2016) showed that the number of DRPs was related to the number of drugs taken by the patients as well as the age of the patients. Our analysis revealed that 92% of patients showed DRPs indicating that patient care requires interdisciplinary collaboration between patients, physicians, and pharmacists.

In our study, we investigated the impact of pharmacist-led and digitally assisted MRs on parameters such as bleeding risks, QoL, adherence and impact on overall DRPs under ambulatory daily standards. As DRPs affect the entire range of drug therapy, the DRPs found in our study group were associated with antithrombotics (26.0%) and co-medication (74.0%).

Similar results were shown in multimorbid patients with type II diabetes (Schindler et al., 2020b), where more DRPs also resulted from co-medication than from the diabetes therapy. Overall, our findings demonstrate that almost half of the DRPs (43.9%) could be reduced through pharmacist-led MRs. Other national studies in Germany, including the ATHINA project, which is still ongoing in several federal states (Seidling et al., 2017; Aktas, 2019; Bitter et al., 2019), were able to demonstrate positive effects of a MR on DRPs, such as a reduction in the frequency and number of DRPs, and on QoL or patient adherence. On the one hand, this could be due to the reduction of patients’ DRPs, such as adverse events. On the other hand, increased patient contact by taking time to explain their medication, possible problems and involving them in their own healthcare might have contributed to an increased feeling of wellbeing and adherence. While the impacts on adherence and QoL showed statistical significance, their clinical relevance for sustained long-term improvements remains unclear, possibly due to the initially high baseline values compared to other studies (Pinto et al., 2011). It should also be noted that the time differences in the follow-up interviews ranged from 1 to 6 months. This may have influenced the results with regard to outcomes such as QoL. In addition, it is difficult to improve the risk of bleeding, especially in the outpatient setting, because it is more often an acute problem that occurs in everyday clinical practice. Nevertheless, this study demonstrated that patient counseling at the community pharmacy resulted in improved medication comprehension, leading to better adherence to the prescribed medication regimen. The difference between the pre- and post-intervention groups was relatively small, with M = 2.4 (adherence), M = 5.7 (QoL, EQ VAS) and M = 0.07 (QoL, EQ Index), respectively. This is consistent with previous studies that have shown only a moderate impact of short-term interventions on patient adherence or QoL (Goh BQ et al., 2014; Verdoorn S et al., 2019). More long-term care studies on this topic need to be conducted to confirm the clinical relevance and sustainability of these findings. Furthermore, the sample size calculation did not rely on the primary study endpoint, which is the reduction of DRPs. It was based on the study by Sennesael et al. Given the relatively limited number of studies that have employed on-site MRs in pharmacy settings, the calculated effect size remains applicable to the primary study endpoint. This assertion finds support in the study of Nasution et al., 2019, who similarly examined DRP reduction in a comparable study involving a total of 45 patients. Nevertheless, the current study shows that pharmacist-led structured MRs in patients with polymedication taking also antithrombotics could be a successful model for managing multiple aspects of medication safety and QoL.

However, a comparable interventional MR study for German antithrombotic patients conducted under real-life conditions in community pharmacies using a DSS is not yet known. The most frequent DRPs were found in relation to the safety of drug therapy, both for antithrombotics and co-medication, which indicates overall drug therapy. Regarding bleeding risks caused by antithrombotic drugs, patients were most frequently at risk of suffering hematoma spontaneously or after minimal trauma (defined by the HEMSTOP questionnaire), whereas major bleedings did not occur. Thus, the bleeding risks were usually potential and not acute. This suggests that there may be problems with side effects due to dosing or drug interactions that increase the risk of bleeding. In our study, the patients did not have any major bleeding, which was also shown by the evaluation of the HEMSTOP-questionnaire.

Schindler et al. (2020a) could show that the most frequent DRPs related to the main medication—in their case, antidiabetic drugs—were due to incorrect dosage. In contrast, in our study, no overdose was observed with antithrombotic medication, whereas drug-drug interactions (13.7%) and adverse side effects (3.4%) were common. However, we did not find an influence of the MR on the reported bleeding risks after the HEMSTOP evaluation. Reasons for this could be the insufficient knowledge of the patient’s records as well as the lack of transparency of patient information or the overall low incidence of bleeding risk in both groups (17.2% at baseline *versus* 14.7% after intervention). Another important factor for the overall low bleeding risk could be a good basal adherence, which could only be moderately improved by further interventions. For example, a retrospective cohort study of patients showed that the risk of hospitalization as one of the primary outcomes in patients with diabetes and hypertension decreased with increasing medication adherence (Sokol MC et al., 2005). The risk of thrombosis (59.0%) was significantly higher than the risk of bleeding (32.8%). This was often due to patients either not taking their antithrombotic medication regularly or deciding to stop taking it entirely. The type of antithrombotic medication clearly plays an important role in the risk of thrombosis, since with VKAs an increased risk of thrombosis develops over a longer period of time after discontinuation of therapy, whereas with DOACs the risk of thrombosis increases rapidly with just one missed dose. It is important to distinguish that good adherence can reduce the risk of thrombosis. On the other hand, poor adherence (with a negative impact on treatment efficacy), may prevent side effects such as an increased risk of bleeding.

Our study also had limitations, mainly of a practical nature. First, local pharmacists in Germany generally do not receive the patients’ laboratory results. In addition, they are usually unaware of the cause, duration, and severity of the patients’ illnesses, unless this has been documented, for example, in a medication plan. If a patient told the pharmacist a diagnosis, a problem related to illness or medication and other facts, it was assumed that this was indeed true, but this could not be routinely checked against the patient’s file, the documented diagnosis, or the laboratory results. Another limitation is due to patients’ reservations about MRs being performed by pharmacists. Some patients consider MRs to be the exclusive responsibility of their general practitioner (GP). This could explain why in 30.3% of the total DRP cases, the status remained unknown. In addition, to assess the long-term impact of DRP reduction on QoL, a longer follow-up period would be needed.

Another interesting factor in the current study was the impact of the DSS software. The performing pharmacists reported efficient support from the DSS, which facilitated the history taking and analysis compared to conventional methods. In particular, the analysis of all possible DPRs, such as correct dosage, contraindications, interactions, patient symptoms as possible side effects or guideline compliance is usually very time-consuming. Other studies without DSS revealed a mean time of 90 min per MR (Seidling et al., 2017). Nevertheless, a direct comparison of the time required for MRs with and without DSS-support needs to be carried out to confirm this assumption. It is important to note that such a DSS-supported MR shows a high number of automatically detected DRPs per patient. These must then be assessed by the pharmacist carrying out the analysis in terms of severity, relevance and priority, also taking into account the personal needs and wishes of the patient. Through direct communication with the patient, the conducting pharmacist effectively identified and prioritized relevant DRPs that were of immediate concern to the patient. It’s important to acknowledge that, given the nature of the task, another pharmacist might not necessarily choose the exact same DRPs with absolute certainty, introducing an element of subjectivity to the selection process. Nevertheless, the pharmacist adopted a systematic approach to discerning critical DRPs, consistently basing the selection on acute patient symptoms and challenges.

In general, there was a notable decrease in DRPs after MR in patients taking antithrombotic drugs. Overall, the number of DRPs was reduced from 234 to 114, indicating a reduction of 120 (51.3%) DRPs. The 61 DRPs associated with antithrombotic medication were reduced by 37 (60.7%) to 24 DRPs, whereas the 173 DRPs linked to co-medication were mitigated by 83 (48.0%) to 90 remaining DRPs. This shows that the interventions resulted in a statistically significant mean reduction from 2.7 DRPs (SD = 1.5) to 1.5 DRPs (SD = 1.2) per patient (1.2 DRPs less per patient, *p* < 0.001). Of all DRPs, 8.1% were partially resolved. In 30.3% of the cases of all DRPs, the status remained unknown, 18.4% of all DRPs could not be resolved and contained the status range “status DRP unknown,” problem/resolution not necessary/possible”; “DRP not resolved, doctor uncooperative,” “DRP not resolved, patient uncooperative.” This could be due to the low number of incidents in both the pre-intervention (average n = 17.2%) and post-intervention groups (average *n* = 14.7%) and the short follow-up interview period of only 1–6 months. The status of the identified DRPs at the end of the study is also a limitation, as it was not possible to verify the extent to which the clinical problems had been finally resolved.

In conclusion, the results of this study demonstrate that pharmacists can effectively support patients receiving antithrombotic therapy and reduce DRPs, also in the context of concomitant polymedication. By offering a valuable MR service as a part of routine care in German community pharmacies, they contribute to an enhanced QoL for multimorbid patients. The promotion of greater acceptance and interprofessional collaboration among physicians, pharmacists, and patients themselves can enhance therapy effectiveness and safety. This aspect should be further developed. Additionally, specialized MR software (DSS) and its integration into daily routines streamline the incorporation of these processes into regular workflow. The DSS utilized in the study provided substantial support to the MR process of the pharmacies.

## Data Availability

The raw data supporting the conclusion of this article will be made available by the authors, without undue reservation.
