# Corrigendum: Impact of a DSS-supported medication review on the safety of drug therapy and quality of life in patients with antithrombotic therapy

**DOI:** 10.3389/fphar.2024.1507320

**Published:** 2024-10-31

**Authors:** Tanja Elnaz Hassanzadeh, Carina Hohmann, Carsten Culmsee

**Affiliations:** ^1^ Pharma4u GmbH, Munich, Germany; ^2^ Institute for Pharmacology and Clinical Pharmacy, Faculty of Pharmacy, University of Marburg, Marburg, Germany; ^3^ Department of Pharmacy, Klinikum Fulda gAG, Fulda, Germany

**Keywords:** drug safety, medication review, community pharmacy, interprofessional collaboration, polypharmacy, quality of life, antithrombotic medication

In the published article, there was an error. The M and SD for the index value were incorrect.

A correction has been made to **3 Results**, *3.3 Quality of life*. This sentence previously stated:

“First, the descriptive part shows a statistically significant improvement of the index value from M = 0.90 (SD = 0.9) to M = 1.1 (SD = 1.2), second, the evaluation of the VAS improved from M = 67.3 (SD = 14.4) to 73.0 (SD = 13.4).”

The corrected sentence appears below:

“First, the descriptive part shows a statistically significant improvement of the index value from M = 0.81 (SD = 0.2) to M = 0.88 (SD = 0.1), second, the evaluation of the VAS improved from M = 67.3 (SD = 14.4) to 73.0 (SD = 13.4).”

In the published article, there was an error. The M value for QoL, EQ Index was incorrect.

A correction has been made to **4 Discussion**, Paragraph 3. This sentence previously stated:

“The difference between the pre- and post-intervention groups was relatively small, with M = 2.4 (adherence), M = 5.7 (QoL, EQ VAS) and M = 0.2 (QoL, EQ Index), respectively.”

The corrected sentence appears below:

“The difference between the pre- and post-intervention groups was relatively small, with M = 2.4 (adherence), M = 5.7 (QoL, EQ VAS) and M = 0.07 (QoL, EQ Index), respectively.”

The authors apologize for these errors and state that these do not change the scientific conclusions of the article in any way. The original article has been updated.

