# Supplementary material for: Impact of a DSS-supported medication review on the safety of drug therapy and quality of life in patients with antithrombotic therapy
Source: Front Pharmacol. 2024 May 23;15:1194201. doi: 10.3389/fphar.2024.1194201 (PMC11153675; doi:10.3389/fphar.2024.1194201)
Supplement: Supplementary file 1 [file Table1.docx]

Supplementary Material

Medication use, classified by ATC-Code level 2
WHO Collaborating Center for Drug Statistics and Methodology, 2019

**Tanja Elnaz Hassanzadeh^1,2^ *, Carina Hohmann^3^ and Carsten Culmsee^2^ ****

^1^Pharma4u GmbH, Ismaninger Str. 3, Rgb., 81675 Munich, Germany

^2^Pharmacology and Clinical Pharmacy, Dept. of Pharmacy, University of Marburg, 35032 Marburg, Germany

^3^Department of Pharmacy, Klinikum Fulda gAG, 36043 Fulda, Germany

*** Correspondence:**Tanja Elnaz Hassanzadeh
hassanzadeh@wir-machen-amts.de

**** Co-Correspondence:**Carsten Culmsee
culmsee@staff.uni-marburg.de

# Supplementary Table

| **ATC-Code Level** | **Medication Class** | **Number of Patients** | **Frequency (%)** |
| --- | --- | --- | --- |
| **B01** | Antithrombotic agents | 87 | 100 |
| **C10** | Agents that influence lipid metabolism | 64 | 73.6 |
| **C09** | Agents with effect on the RAAS system | 61 | 70.1 |
| **C07** | ß-adrenoceptor antagonists | 40 | 46.0 |
| **N02** | Analgesics | 38 | 43.7 |
| **A02** | Remedy for acid-related diseases | 34 | 39.1 |
| **C03** | Diuretics | 28 | 32.2 |
| **C08** | Calcium channel blocker | 26 | 29.9 |
| **A10** | Antidiabetics | 25 | 28.7 |
| **H03** | Thyroid therapy | 24 | 27.6 |
| **N06** | Psychoanaleptics | 22 | 25.3 |
| **A11** | Vitamins | 21 | 24.1 |
| **A12** | Minerals | 21 | 24.1 |
| **R03** | Remedy for obstructive respiratory diseases | 20 | 23.0 |
| **G04** | Urologics | 16 | 18.4 |
| **N05** | Psycholeptics | 12 | 13.8 |
| **M04** | Gout remedies | 11 | 12.6 |
| **A06** | Agents against constipation | 9 | 10.3 |
| **C01** | Cardiac therapy | 9 | 10.3 |
